# First insights into the gut microbiomes and the diet of the *Littorina* snail ecotypes, a recently emerged marine evolutionary model

**DOI:** 10.1111/eva.13447

**Published:** 2022-07-24

**Authors:** Marina A. Z. Panova, Marina A. Varfolomeeva, Elizaveta R. Gafarova, Arina L. Maltseva, Natalia A. Mikhailova, Andrei I. Granovitch

**Affiliations:** ^1^ Department of Marine Sciences‐Tjärnö University of Gothenburg Gothenburg Sweden; ^2^ The Centre for Marine Evolutionary Biology CeMEB University of Gothenburg Gothenburg Sweden; ^3^ Department of Invertebrate Zoology St. Petersburg State University St. Petersburg Russia; ^4^ Centre of Cell Technologies Institute of Cytology RAS St. Petersburg Russia

**Keywords:** ecological divergence, host‐microbiome interaction, intertidal, *Littorina*, microbiome

## Abstract

Microbes can play a prominent role in the evolution of their hosts, facilitating adaptation to various environments and promoting ecological divergence. The Wave and Crab ecotypes of the intertidal snail *Littorina saxatilis* is an evolutionary model of rapid and repeated adaptation to environmental gradients. While patterns of genomic divergence of the *Littorina* ecotypes along the shore gradients have been extensively studied, their microbiomes have been so far overlooked. The aim of the present study is to start filling this gap by comparing gut microbiome composition of the Wave and Crab ecotypes using metabarcoding approach. Since *Littorina* snails are micro‐grazers feeding on the intertidal biofilm, we also compare biofilm composition (i.e. typical snail diet) in the crab and wave habitats. In the results, we found that bacterial and eukaryotic biofilm composition varies between the typical habitats of the ecotypes. Further, the snail gut bacteriome was different from outer environments, being dominated by Gammaproteobacteria, Fusobacteria, Bacteroidia and Alphaproteobacteria. There were clear differences in the gut bacterial communities between the Crab and the Wave ecotypes as well as between the Wave ecotype snails from the low and high shores. These differences were both observed in the abundances and in the presence of different bacteria, as well as at different taxonomic level, from bacterial OTU's to families. Altogether, our first insights show that *Littorina* snails and their associated bacteria are a promising marine system to study co‐evolution of the microbes and their hosts, which can help us to predict the future for wild species in the face of rapidly changing marine environments.

## 1 INTRODUCTION

Microbes can play a prominent role in the evolution of their hosts, facilitating adaptation to various environments and promoting divergence and speciation. Due to their ability to change rapidly and their wide metabolic scope, microbiomes can help macro‐organisms to adapt to rapidly changing environments (Alberdi et al., 2016). Gut microbiomes are especially important for their hosts for performing many fundamental biological functions (Macke et al., 2017; Shapira, 2016). Gut microbiome includes a core microbiome, adapted to the host, and a facultative part, depending on the environment (Shapira, 2016). The gut microbial assemblage possesses high metabolic diversity, and there are numerous examples on how this diversity is utilized by the host. For example, bacteria perform the first digestion steps of complex polysaccharides in the rumen of herbivorous mammals that lack the ability to digest those substrates themselves (Flint et al., 2008). Bacteria can also degrade toxic secondary metabolites that plants produce in defense against the herbivores, as the caffeine detoxification by *Pseudomonas* in the guts of the coffee insect pest (Ceja‐Navarro et al., 2015). Further, bacteria can supply their host with nutrients that are absent in the diet, e.g. *Buchnera* provides essential amino acids for the aphids (Hansen & Moran, 2011), or can metabolize the waste compounds, e.g. *Clostridia* helps termites to recycle uric acid (Thong‐On et al., 2012). Another important function of the gut symbionts is protection from other, pathogenic, microorganisms through a direct competition or by modulating the host immune response (Kamada et al., 2013).

Not surprisingly, the divergence between species and populations is often associated with divergence of the gut microbiomes, as shown for different species and population of termites (Hongoh et al., 2005). In the Baltic sand lances gut microbiome variation reflects the salinity gradient as well as genetic divergence between the species and populations of the host (Fietz et al., 2018). Thus, ecological divergence is often associated with divergence of the host microbiomes. Microbiome variability may even be a driver in the evolution of reproductive barriers. For example, in *Drosophila* species the composition of the gut bacteria depends on the food source (e.g. ecology of the host) and has a strong effect on the mate choice through the changes of the mating cues, cuticular hydrocarbons. As a result, flies show a clear mating preference for the individuals grown on a similar diet (Lizé et al., 2013). Another example is *Costelytra* insects, where microbes them‐selves produce the pheromones (Hoyt et al., 1971). Thus, microbes have a prominent effect on evolution of their hosts, facilitating adaptation to new ecological niches and even driving divergence between the populations by changing mating preferences.

Most of our knowledge on the composition and the role of host‐associated microbiomes so far came from the mammals (Krajmalnik‐Brown et al., 2012; Ley et al., 2008) and insects (Douglas, 2014; Engel & Moran, 2013). Recently, the scope had expanded to other species, including marine mollusks (Choi et al., 2021; Fernandez‐Piquer et al., 2012; Gobet et al., 2018; Song et al., 2018), and among them the genus *Littorina* (Maltseva et al., 2021; Neu et al., 2019). These studies described host‐associated microbiomes, and also significant differences between closely related species of the snails (Maltseva et al., 2021). Further, a recent study discusses the possible role of microbes in the snail genome evolution (Maltseva et al., 2022).

*Littorina* snails are one of the well‐established model systems in evolutionary biology, as it includes groups with divergence from millions of years to recent post‐glacial ecotypes and living side by side in the intertidal (Maltseva et al., 2020; Panova et al., 2014; Reid, 1996; Reid et al., 2012). The majority of studies has focused on the genomic architecture behind a very recent divergence of the Crab and Wave ecotypes of *L. saxatilis* (Faria et al., 2019; Johannesson et al., 2017; Morales et al., 2019; Westram et al., 2018). In Sweden, the Crab and Wave ecotypes inhabit sheltered boulder beaches and the wave‐exposed cliffs respectively; the crab predation and dislodgement by waves are assumed to be the main selective agents, responsible for variation in size, shell shape and shell sickness of the snails (Le Pennec et al., 2017; Westram et al., 2018, 2021). The two habitats, however, most likely differ in many other aspects, such as desiccation time, temperature regimes, salinity fluctuations, etc. These abiotic factors in turn affect the assemblages of both the macro‐ and microorganisms. For example, Pfister et al. (2014) showed differences in the microbiome composition associated with different substrates and intertidal macro‐organisms, e.g. rock surface, mussel shells or red algae. Such differences can affect both snails' diet, since *L. saxatilis* is a micro‐grazer feeding on the biofilm on the surface of rocks (Fretter & Graham, 1962; Reid, 1996 and references therein), and the gut microbiome of the snails if the two ecotypes live on different diets and encounter different bacteria in their environment. In addition, the Wave ecotype lives along the vertical gradient on the cliffs, and a few studies showed genetic adaptation of snails to low and high intertidal zones (Johannesson et al., 1995; Morales et al., 2019; Panova & Johannesson, 2004). Vertical zonation on the rocky shores is one of the strongest environmental gradients in the nature and likely to shape the microbiome composition. For example, the tidal zonation has been shown to affect bacterial biofilms on the shores in Chile (Arboleda‐Baena et al., 2021) and microbiomes of the sponges living in the subtidal and intertidal (Weigel & Erwin, 2017).

This study aims to investigate microbe composition in the environment of the Crab and Wave ecotypes as well as the microbiome associated with the snails to answer the following questions:

1. Are there differences in the taxonomic composition of the microbial biofilm communities (i.e. the snail diet) between the typical habitats of the snail ecotypes?
2. Do the snails host a specific gut microbiome, significantly different from the outer environment?
3. Are there differences in the composition of the gut microbiome between the snail ecotypes?

**FIGURE 1:**
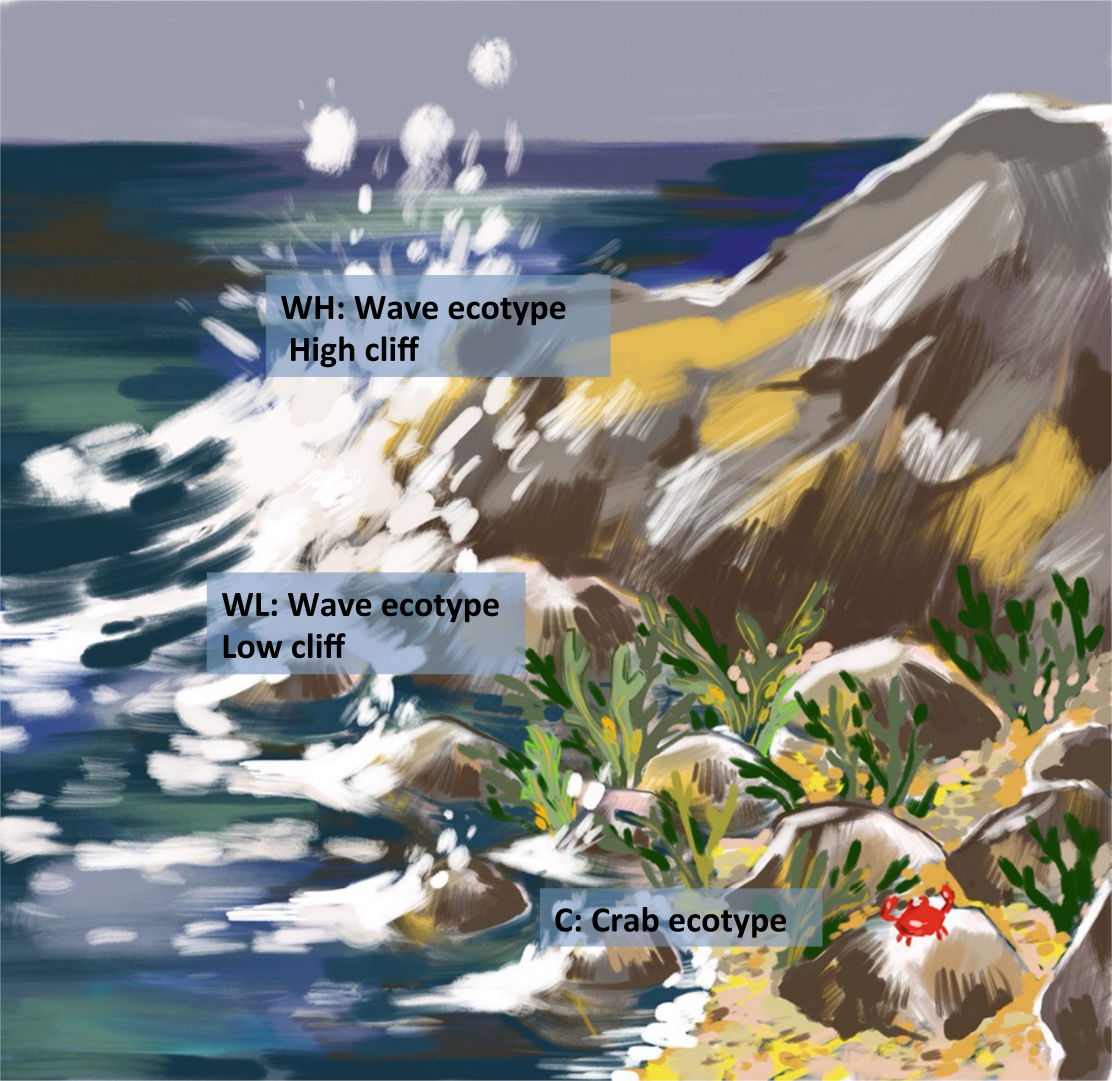
The *Littorina saxatilis* ecotypes in their typical habitats on the Swedish west coast (the author of artistic drawing: Maria Sologub).

In our study, we included three types of environments, common on the North Atlantic rocky shores in Sweden: (1) a sheltered boulder beach, the typical environment for the Crab ecotype; (2) a lower part of the exposed cliffs and (3) a high part of the exposed cliffs, the latter two inhabited by the Wave ecotype (Figure 1).

## 2 MATERIALS AND METHODS

### 2.1 Sample collection

Samples were collected on two islands close to the Tjärnö Marine Laboratory: Arsklåvet (58°49′52.6″N 11°04′00.8″E) and Ramsö (58°49′58.1″N 11°08′03.5″E) in August 2018. At each site we collected *L. saxatilis* snails within a few square meters are in the typical Crab and Wave habitats: a boulder beach and a cliff. The Wave ecotype snails were collected separately from the low and the high parts of the cliff. The snails were brought alive to the laboratory and dissected within the same day. The mid‐ and hindgut parts from five randomly selected individuals were pooled, and three pooled samples were prepared for each site and the species. These samples were used to characterize gut microbiome and gut content of the snails. Pooling was done in order to obtain enough biological material for analyses and to reduce the biological variation between the samples. These samples were analyzed both for bacterial and eukaryotic DNA with the 16S and 18S markers, respectively.

In addition to the gut samples we collected samples from the body surface of snails. To do this, cephalic tentacles of all snails used for the gut samples (15 individuals per group) were combined together. These samples were not intended for analyses of the skin microbiomes but merely to control that microbiome communities within the gastrointestinal tracts of the snails are different from those outside. The skin samples were only analyzed for bacterial composition using the 16S maker.

The environmental samples were obtained by scraping of natural substrates inhabited by periwinkles by a sterile scalpel in several places within the snail collection area. In each locality we collected three samples from each type of environment: high cliff, lower cliff and boulder surfaces. These samples were placed in 70% ethanol and kept frozen until DNA extractions. The environmental samples were analyzed both for bacterial and eukaryotic DNA. Altogether, we collected 42 samples of the snail ecotypes and their environments from two localities. A negative laboratory control, produced by wiping Petri dishes used for the mollusk dissection with sterile tissue was processed in the same way as the samples.

### 2.2 Library preparation and sequencing

DNA extractions were done with the DNeasy PowerSoil kit (QIAGEN) following the manufacturer's protocol. DNA quality and quantity was accessed with the Nanodrop spectrophotometer and Qubit fluorometer.

The library preparation process followed the online protocol https://github.com/EnvGen/LabProtocols/blob/master/Amplicon_dual_index_prep_EnvGen.rst. The metabarcoding fragment of 16S rRNA gene was amplified using universal Eubacteria primers 314F and 805R (Klindworth et al., 2013), extended with adapters for the index PCR. PCR reactions were performed in 20 μl volume, containing 10 μl KaPa HiFi HotStart ReadyMix 2x (Roche), 1 μl of 10 mM forward and reverse primers, 0.5 μl of bovine serum albumin (20 mg/ml), 2.5 μl of PCR‐grade water and 5 μl of DNA template (1 ng/μl). PCR cycling was as follows: 98°C for 2 min; 25 cycles at 98°C for 20 s, 54°C for 20 s, 72°C for 10 s; 72°C for 2 min.

The metabarcoding fragment of 18S rRNA gene was amplified using universal eukaryote primers EUK1181 and EUK1624 (Wang et al., 2014), extended with adapters for the index PCR. To prevent the amplification of the snail DNA we developed a blocking primer GTGTACAAAGGGCAGGGACG with C3‐spacer, following the strategy suggested by Vestheim and Jarman (2008). PCR reactions were performed in 25 μl volume, containing 12.5 μl KaPa HiFi HotStart ReadyMix 2x (Roche), 0.75 μl of the 10 mM forward and reverse primers, 4 μl of the 10 mM blocking primer, 0.5 μl of bovine serum albumin (20 mg/ml), 5.5 μl of PCR‐grade water and 1 μl of DNA template (1 ng/μl). PCR cycling was as follows: 95°C for 5 min; 35 cycles at 95°C for 30 s, 58°C for 30 s, 50°C for 50 s, 72°C for 60 s; 72°C for 5 min (each cycle included an extra step for annealing of the blocking primer at 58°C).

PCR products were visualized on the agarose gel to confirm successful amplification and cleaned with AMPure XP beads (Beckman Coulter) following the Illumina amplicon sequencing protocol (https://support.illumina.com/documents/documentation/chemistry_documentation/16s/16s‐metagenomic‐library‐prep‐guide‐15044223‐b.pdf). The second, index, PCR was performed in 28 μl volume, containing 14 μl KaPa HiFi HotStart ReadyMix 2x (Roche), 1 μl of index 1 and index 2 primers and 12 μl of cleaned inner PCR products at 2 ng/μl. PCR cycling was as following: 98°C for 2 min; 8 cycles at 98°C for 20 s, 62°C for 30 s, 72°C for 30 s; 72°C for 2 min. PCR products were cleaned with AMPure XP beads (Beckman Coulter) following the Illumina protocol. The concentrations of the cleaned PCR were measured with Qubit fluorometer and PCR products were pooled in equimolar concentrations. The samples were sequenced on the Illumina MiSeq v3‐600 (2x300) platform by the National Genomic Infrastructure (NGI) in Sweden, 96 samples per run. The raw data are deposited to the NCBI archive, BioProject PRJNA778532.

### 2.3 Data processing and bioinformatics analysis

The reads were demultiplexed by the National Genomic Infrastructure (NGI). The quality of the raw data was assessed with *FastQC* (https://www.bioinformatics.babraham.ac.uk/projects/fastqc/). Low‐quality nucleotides and adapter sequences were removed with *cutadapt* (Martin, 2011). Further processing was done using *mothur* pipeline (Kozich et al., 2013; Schloss et al., 2009). Forward and reverse reads were joined into contigs; after that the contigs with ambiguous bases, with homopolymer regions longer than 8 nucleotides and longer than the expected fragment length were removed.

For the 16S data, unique sequences were aligned to the SILVA 16S rRNA reference database v132, December 2017 (Quast et al., 2013; Yilmaz et al., 2014) and sequences producing poor alignments as well as chimeric sequences and singletons were removed. Remaining sequences were classified at the domain level and Eukaryotes, Archaea, chloroplasts, mitochondria and unclassified sequences were removed. The remaining bacterial 16S rRNA sequences were clustered in operational taxonomic units (OTUs) at 97% similarity level, which roughly corresponds to the difference between bacterial genera, and taxonomy was assigned using the Bayesian RDP classifier (Wang et al., 2007). Finally, the number of reads in all samples was subsampled to the smallest sample size and rare OTUs (occurring at <100 reads in the total dataset) were removed. The negative control was processed together with the samples. After filtering it contained a low number of sequences that were assigned to few bacteria, commonly associated with humans and laboratory environments and therefore was excluded from further analyses.

For the 18S data, unique sequences were classified using the PR2 database (del Campo et al., 2018; Guillou et al., 2013). This database was chosen because it contains the species‐level taxonomy and left far less unclassified sequences compared to the SILVA 18S. Mollusca, chloroplasts, mitochondria and unclassified sequences were removed. Despite good results in the initial PCR test, the blocking primer did not prevent the host DNA amplification and the majority (approximately 90%) of reads in the gut samples came from the snails. This resulted in much lower sample sizes for the gut samples compared to the environmental samples. Therefore, the number of reads in the 18S dataset strongly varied (approximately 50–1000 reads per sample in the snail samples; 10,000–20,000 reads per sample in environmental samples). We constructed rarefaction curves in the *vegan* package to check whether the sequencing depth adequately represented microbial communities. Since the minimal number of reads was small, the subsampling was not done to avoid sample size effects. Rare OTUs (occurring at <100 reads in the total dataset) were removed. The negative control did not contain any sequences after filtering.

### 2.4 Statistical analyses

Data analysis was performed in R (R v.4.1.2; R Core Team, 2021). 18S and 16S datasets were analyzed separately. To describe alpha diversity in microbiome samples, we calculated Shannon‐Wiener diversity index using the formula $H^{\prime }=-\sum_{i=1}^{S}p_{i}\log_{e}\;p_{i}$, where *p*_*i*_ is the proportion of OTUs of a particular taxon S (Shannon, 1948).

Microbiome samples were ordinated using nonmetric multidimensional scaling (nMDS). For this analysis, square‐root transformed OTU abundances were wisconsin‐standardized (first, OTU abundances were standardized by their maxima, then the samples were divided by the total abundance of all OTUs in each sample) and the Bray–Curtis dissimilarity matrix was computed (Bray & Curtis, 1957). NMDS was performed with multiple random starts to find the best two‐dimensional solution. The quality of ordination was assessed using the *stress1* value (Kruskal, 1964). The analysis was carried out using the *vegan* package (Oksanen et al., 2020).

The permutation analysis of variance (PERMANOVA; Anderson, 2001) was done to test for differences in the microbiome composition between groups of samples, independently for 16S and 18S data. First, microbiomes of different microhabitats were compared. The model included two discrete predictors: microhabitat (boulders, high and low shore cliffs) and site (Arsklåvet and Ramsö), and interaction of these factors. Second, gut microbiomes were compared between *L. saxatilis* ecotypes by a two‐way ANOVA with factors ecotype (Crab, Wave‐High, Wave‐Low), locality and their interaction. The distance matrices for PERMANOVA were preprocessed in the same way as for nMDS ordination. PERMANOVA assumptions were checked using “betadisper” function of the *vegan* package and were met in all cases. Tests were performed on 10,000 permutations. Post‐hoc pairwise comparisons were carried out to test differences between the groups. *p*‐values were corrected for multiple testing using Holm‐Bonferroni method (Holm, 1979).

The taxonomic composition and relative abundance of dominant taxa were plotted using the *Fantaxtic* R package (Teunisse, 2018) and the *KronaTools* Python scripts v2.8 (Ondov et al., 2011). Venn diagrams were constructed using the *eulerr* R package (Larsson, 2018). Other plots were built using the *ggplot2* package (Wickham, 2016).

## 3 RESULTS

### 3.1 Bacterial diversity and differentiation in the different environments and in the snail ecotypes

The number of clean 16S reads after filtering was 49,106–277,766 per sample, consequently all samples were subsampled to 49,106 reads. In total, we detected 12,566 OTUs; after removing OTUs represented by <100 reads the dataset contained 1233 OTUs. The alpha‐diversity was high in the all environmental and most of the snail‐associated microbiome samples (Figure S1). The mean numbers of OTUs in the environmental, body surface and gut samples were 350, 394 and 359 respectively.

**FIGURE 2:**
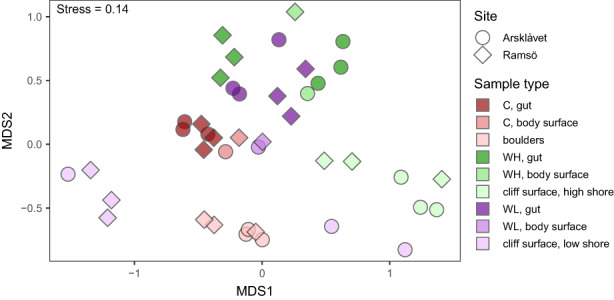
Nonmetric multidimensional scaling (nMDS) ordination of bacterial communities in the environment, on the body surface, and in the gut of snails. The analysis was based on the matrix of pairwise Bray‐Curtis dissimilarities between square‐root transformed and Wisconsin‐standardised OTU abundance in samples. Shape indicates collection site; main colour indicates sample groups and colour shade indicates sample type within each group (environment, body surface and gut). Letters indicate snail ecotype: C, Crab ecotype; WH, Wave ecotype, high shore; WL, Wave ecotype, low shore.

In the ordination plot there was a clear separation between the environmental and the snail‐associated microbiomes but not between the two locations (Figure 2). Within the environmental samples the boulders formed one distinct group. The cliff samples had higher variation; one group contained all the low shore samples plus two of the high shore samples and another group was formed by the remaining four high shore samples. These results were confirmed by the neighbour‐joining clustering (Figure S2a). In the PERMANOVA results, biotope, locality and their interaction had significant effects (*p* = 0.0001, 0.0007 and 0.01, respectively). The post‐hoc comparisons were done combining the two localities due to the low number of replicates per locality. They showed significant differences between all three types of habitats: boulders, the low shore cliffs and the high shore cliffs (*p* = 0.003–0.46). In the analysis of the gut microbiome composition of the *L. saxatilis* ecotypes (Crab; Wave‐Low and Wave‐High) there were significant effects of the ecotype, locality and their interaction (*p* = 0.0001, 0.035 and 0.001 respectively). In the post‐hoc comparisons, there were significant differences between all three pairs of the ecotypes (*p* = 0.003–0.006). In the ordination plot and the neighbour‐joining clustering analyses the gut samples from the Crab‐ecotype formed one distinct cluster while the gut samples from the Wave ecotypes showed more variation and no clear separation between the high and low shore (Figure 2 and Figure S2b).

### 3.2 Dominant and specific bacteria in the different environments and in the snail ecotypes

**FIGURE 3:**
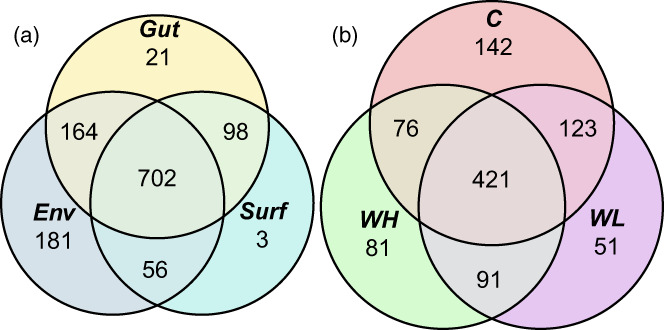
Venn diagrams of the bacterial OTUs present in the different types of samples (a: Environment/body surface/guts) and the guts of the different ecotypes of *Littorina saxatilis* (b: C, Crab ecotype; WH, Wave ecotype, high shore; WL, Wave ecotype, low shore).

Most of the OTUs were present in all three types of the samples. However, some OTUs were found only in the environment or only in the snail guts; very few OTUs were specific for the body surface samples (Figure 3a; see also an interactive Krona‐diagram in File S1). Among the bacteria living in the intertidal environment but not associated with snails there were several genera from Saprospiraceae (*Lewinella*, *Rubidimonas*, *Portibacter* and others), Flavobacteriaceae (*Muriicola*, *Ulvibacter* and others), Desulfobulbaceae, unclassified Gammaproteobacteria, Cyclobacteriaceae, Rhodobacteraceae plus some other families represented by one to few genera.

The full lists of OTUs are available in Table S1. The bacteria found only in the snail guts were *Mycoplasma*, *Enterococcus*, *Halomonas*, *Pseudomonas* and *Persibacter*, as well as unclassified members of Planctomycetes, Rhodobacteraceae, Pirellulacea and Devosiaceae.

**FIGURE 4:**
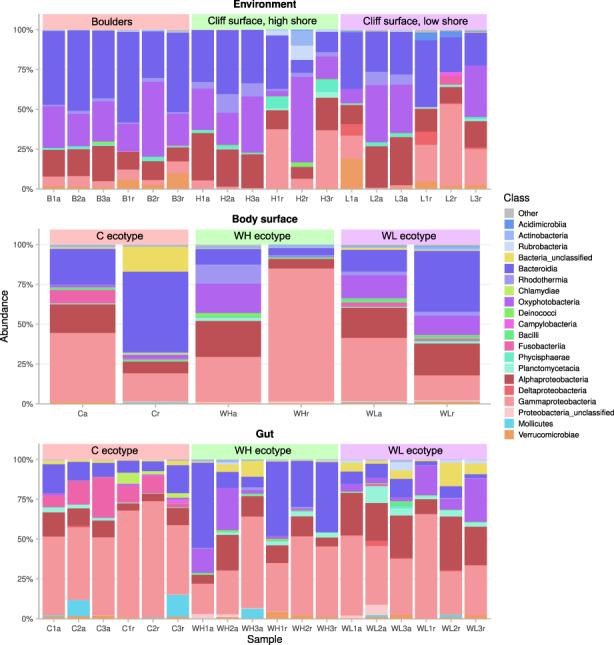
Taxonomic composition of bacterial communities at the class level in the biofilms, snail body surface and snail’ guts. Colours indicate 20 most abundant OTU classes in each sample; all other OTUs are shown in grey. *Littorina saxatilis* ecotypes are designated by C, Crab ecotype; WH, Wave ecotype, high shore; WL, Wave ecotype, low shore).

At the higher taxonomic level, the abundance of dominating bacteria differed between the environment and the snail‐associated samples. For example, the dominating classes in the environmental samples were Bacteroidia, followed by Oxyphotobacteria, Alphaproteobacteria and Gammaproteobacteria (Figure 4, File S1). In the snail gut, the class Gammaproteobacteria was the most abundant, followed by Fusobacteria, Bacteroidia and Alphaproteobacteria (Figure 4; File S1). The composition of the snail body surface microbiome was in‐between the environmental and the gut microbiomes, e.g. Bacteroidia were less abundant than in the environment but more common than in the guts and Gammaproteobacteria were more abundant than in the environment but less common than in the guts (Figure 4). Relatively high numbers of bacteria in some snail samples could not be classified even at the high taxonomic levels (File S1).

Taxonomic composition of the snail gut microbiome differed between the snail ecotypes both due to the abundance level of shared bacteria and due to some host‐specific bacteria. In the Crab‐ecotype of *L. saxatilis* the most common bacteria were *Vibrio*, Proteobacteria (31%), *Psychrilyobacter*, Fusobacteria (10%) and *Catenococcus*, Proteobacteria (9%). In the Wave‐Low shore ecotype the most common were again *Vibrio* (11%), *Catenococcus* (9%) and an unclassified Rhizobiaceae, Proteobacteria (8%). In the Wave High‐Shore ecotype the most common bacteria were an unclassified Enterobacteriaceae, Proteobacteria (18%), an unclassified Flavobacteriaceae, Bacteroidetes (15%) and *Pleurocapsa*, Cyanobacteria (7%); *Vibrio* and *Catenococcus* were present at 6 and 5% respectively.

The three ecotypes of *L. saxatilis* shared a large part of their gut microbes (421 OTUs) but there were also bacteria specific for each ecotype (Figure 3b). 142 OTUs occurred only in the guts of Crab‐ecotype snails and 223 – only in the guts of the Wave‐ecotype snails; there were also OTU's specific for the Wave snails from the high or low shores (Figure 3b). Much of these differences were related to the family level, i.e. 34 bacterial families were specific for the guts of the Crab ecotype, 18 – for the guts of the Wave‐High ecotype and 10 – for the guts of the Wave‐Low ecotype. Eight bacterial families were common in the guts of all the ecotypes: Flavobacteriaceae, Saprospiraceae, Rhodobacteraceae and Pirellulaceae. However, in the different snail ecotypes these bacterial families were represented by different genera.

### 3.3 Eukaryotic profiles in the environmental and the snail gut samples

We detected 1273 eukaryotic OTUs; 236 OTUs were left after removing OTUs represented by <100 reads in the total dataset. The amount of data from the snail gut samples was limited due to insufficient blocking of the host DNA amplification. However, the sequences that were retained represent the most common species in the samples. Further, rarefaction analysis showed that only one gut sample under‐sampled true taxonomic diversity (Figure S3). Still, taxonomic profiles of eukaryotic DNA in the snail guts may not be complete and should be regarded with caution.

**FIGURE 5:**
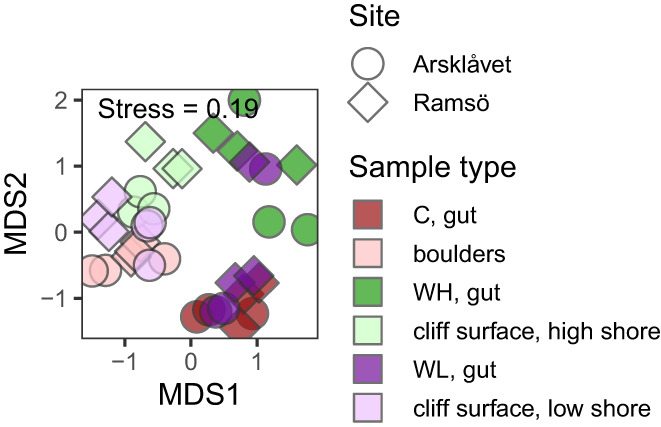
Nonmetric multidimensional scaling (nMDS) ordination of eukaryotic communities in the environment and in the gut of *Littorina* snails. The analysis was based on the matrix of pairwise Bray‐Curtis dissimilarities between square‐root transformed and Wisconsin‐standardised OTU abundance in samples. Shape indicates collection site; main colour indicates sample groups and colour shade indicates sample type within each group (environment vs. gut). Letters indicate snail ecotype: C, Crab ecotype; WH, Wave ecotype, high shore; WL, Wave ecotype, low shore.

There was no obvious trend for higher alpha‐diversity in a particular sample type (Figure S4). Overall patterns were similar to the bacterial beta‐diversity: a clear separation between the environmental and the snail‐associated samples; *L. saxatilis* Crab‐ecotypes are separated from *L. saxatilis* Wave‐High ecotype; and *L. saxatilis* Wave‐Low ecotype being in‐between the Crab and the Wave‐High (Figure 5; see also Figure S5a,b for the neighbour‐joining clustering diagrams).

In the PERMANOVA analyses comparing the different environments, there were significant effects of environment, locality and their interactions (*p* = 0.0001, 0.008 and 0.0001 respectively). The difference between the high shore and the low shore cliffs was significant before correction for multiple testing (*p* = 0.046 before, *p* = 0.138 after correction); the difference between the cliffs and the boulders was not significant. In the clustering diagrams, the boulder samples from the two localities clustered separately (Figure S5a) but this could not be tested statistically due to insufficient number of replicates. The comparison of the eukaryotic species in the snail guts showed significant effect of ecotype and locality (*p* = 0.009 and 0.005), but pairwise comparisons pooling the two localities were non‐significant.

Much of the DNA both in the environmental samples and in the snail guts came from the macro‐organisms, e.g. red crustose algae (Hildenbrandiales), green algae (Ulotrichales) and small maxillopod crustaceans. It could originate from environmental DNA or from small propagules and larvae. The other common groups were marine filamentous fungi (Pezizomycotina) and protists (ciliates, dinoflagellates, bicosoecids), see an interactive Krona diagram in File S2. Of the major components of intertidal microphytobentos we found many diatoms (*Cylindrotheca*, *Licmophora*, *Cyclophora*, *Tabularia*, *Nitzschia*, etc.).

**FIGURE 6:**
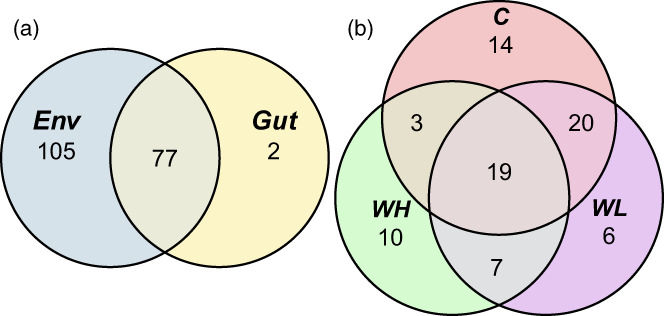
Venn diagrams of the eukaryotic OTUs present in the different types of samples (a: Environment vs. guts) and the guts of the different ecotypes of *Littorina saxatilis* (b: C, Crab ecotype; WH, Wave ecotype, high shore; WL, Wave ecotype, low shore).

**FIGURE 7:**
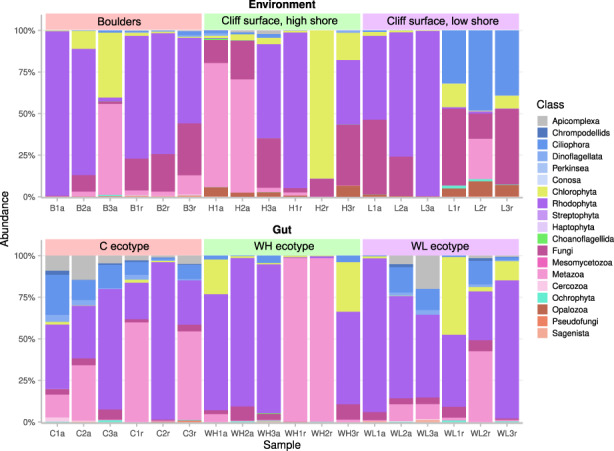
Taxonomic composition of eukaryotic communities at the class level in the biofilms and snail guts. Colours indicate 20 most abundant OTU classes in each sample; all other OTUs are shown in grey. *Littorina saxatilis* ecotypes are designated by C, Crab ecotype; WH, wave ecotype, high shore; WL, wave ecotype, low shore.

Most of the taxa found in the snail guts came from the environments (Figure 6). However, the proportions of some groups (e.g. Chlorophyta, Ciliophora, Dinoflagellata) appear to be different in the snail guts and their environments (Figure 7, Table S2). Surprisingly, much of DNA in some snail gut samples had an animal origin, e.g. from *Tigriopsus* copepods (Figure 7, Table S2). In general, the variation in 18S profiles between the replicate samples (both from environments and snail guts) appears to be much higher than between 16S profiles (Figure 7 as compared to Figure 3).

Cyanobacteria are the second most important component of the intertidal epilithic biofilms after diatoms and therefore should be considered as an important part of the snail diet along with the eukaryotic organisms. Cyanobacteria composed 19%–26% of all bacteria in the rock surface biofilms with Nostocales and Phormodesmirales being most common groups (Oxyphotobacteria in Figure 4; File S1). Assuming that cyanobacteria are a part of the snail diet, they composed ≤1% of all bacteria in the gut samples of the Crab‐ecotype of *L. saxatilis*, while in the guts of the Wave‐ecotype 8%–11% of all bacteria were cyanobacteria (Figure 4 and File S1).

## 4 DISCUSSION

It is becoming more and more evident that microbes can greatly enhance the adaptive potential of their host. By providing various metabolic advantages they can facilitate the adaptation to new environment or even promote changes in ecological preferences of the host. Studying host‐associated microbiome is therefore imperative to understand and predict the evolutionary responses of the wild animals to a changing environment (Bahrndorff et al., 2016; Fietz et al., 2018; Hird, 2017). Indeed, it has been argued that co‐existence with microbial symbionts was an important factor in the evolution of vertebrates, and the understanding of animal biology is only possible in connection to their bacterial symbionts (McFall‐Ngai, 2007; McFall‐Ngai et al., 2013). A potential for rapid changes in the host‐associated microbiome and its profound effect on the host phenotype (Alberdi et al., 2016) makes the microbes an important and still very much hidden player in the adaptation of macro‐organisms to rapid environmental changes.

The Crab and Wave ecotypes of *L. saxatilis* represent a striking example of a rapid ecological divergence, repeatedly occurring in nature. In this study, we provide a first insight into the host and environmental microbiomes associated with the snail ecotypes, suggesting that the microbe can play an important role in the snails' adaptation to different environments. In the present study we found profound differences between the bacterial communities associated with the different ecotypes, as well as in the environmental microbiomes in the typical ecotype habitats of the ecotypes. Further, we found some support that the diet of ecotypes may be different. These findings and their potential relevance to the evolution of the snail ecotype are discussed below.

### 4.1 Environmental and snail‐associated microbiomes

We found high bacterial diversity both in the environmental and host microbiomes, 12,566 OTUs in total. Diverse bacterial communities are associated with other marine invertebrates (Moisander et al., 2015; Weigel & Erwin, 2017; Wenzel et al., 2018), and this may reflect the vast diversity of marine bacteria in general (Glöckner et al., 2012). In the environmental samples the dominant phyla were Proteobacteria and Bacteroidetes, which is typical for marine environment (Bolhuis & Cretoiu, 2016; Gilbert et al., 2009; Wenzel et al., 2018). In addition, Cyanobacteria, which in our study were the third dominant group, are an important component of intertidal biofilm communities (Decho, 2000; Thompson et al., 2004).

There were some differences between the bacterial communities in the biofilm on the boulder beaches and lower and upper parts of the cliffs, in agreement with other studies on intertidal zonation of microbial communities (Arboleda‐Baena et al., 2021; Weigel & Erwin, 2017). In the previous study we found regional differences in the intertidal microbiomes between Norway and Sweden (Maltseva et al., 2021). The present study was conducted at the local scale and there were no significant differences between sites that were only few km apart.

There was a clear separation between the environmental microbiomes and the bacteria associated with the snail host, which agrees with the recent finding for the Pacific *L. keenae* (Neu et al., 2019) and several Atlantic *Littorina* species (Maltseva et al., 2021). Similar to the studies above we found some bacterial families exclusively associated with the snail host but the majorities of differences were at the abundance level. The composition of the microbiome on the body surface of the snails was intermediary to the biofilm and gut samples, likely including both the microbes currently present in the environment and those always associated with the snails. Altogether these findings provide strong evidence that there is a host‐specific microbiome associated with *Littorina* snails. The snail‐associated microbiomes are likely to be formed under strong influence of the snail environment. This might be through an uptake of bacteria present in the environment, that serves a medium for development of a bacterial community in the gut, or through a regulation of the bacteriome by the environmental conditions, as was shown for microbiomes associated with subtidal and intertidal populations of a marine sponge (Weigel & Erwin, 2017). Notably, the differences between the boulder beaches – low cliffs – high cliffs are also reflected in the eukaryotic assemblages described below.

Some of the most common bacteria associated with *Littorina* were previously found in the guts of other marine invertebrates. Thus, *Vibrio* was also observed in the tunicate *Ciona* (Utermann et al., 2020), the isopod *Jaera* (Wenzel et al., 2018) and in other marine invertebrates (Pfister et al., 2014). *Psychrilyobacter* was also found in the guts of many marine invertebrates, including several gastropod species (Alain et al., 2012; Dishaw et al., 2014; Gobet et al., 2018; Song et al., 2018). The genus *Catenococcus* (Vibrionaceae), common in the guts of the *L. saxatilis* Crab ecotype, has not been, to our knowledge, reported as a dominant bacterium in the guts of marine invertebrates.

We found the significant differences in the microbiomes associated with the *L. saxatilis* ecotypes. There were large differences were between the Crab and the Wave ecotypes; however, there were also differences in microbiomes of the Wave snails from low and high shore. The latter results agree with other studies showing the strong effect of the vertical shore gradients both on environmental bacterial communities and microbiomes of intertidal macro‐organisms (Arboleda‐Baena et al., 2021; Weigel & Erwin, 2017). This also supports the view that in many aspects, including microbiomes, the Wave ecotype snails should not be regarded as one homogeneous group.

Bacterial diversity associated with the snail ecotypes can be analyzed at the levels of presence or abundances and also at the different taxonomic levels. At the presence level, we found more than 300 OTUs present in only one ecotype, when the Wave low and Wave high ecotypes were considered separately. Some differences in microbiome composition were already at the family level, i.e. 62 bacterial families were present in only one ecotype. Other families (e.g. Flavobacteriaceae, Saprospiraceae, Rhodobacteraceae and Pirellulaceae) were associated with all snails but represented in the ecotypes by different genera. At the same time, some common marine bacteria such as *Vibrio*, *Psychrilyobacter* and *Catenococcus*, were detected in the guts of all snails but at very different abundance level. The first taxonomic description of the snail‐associated microbiomes presented here do not allow us to determine which of these differences in the microbiome composition are important for the host evolution. The present findings however indicate a high potential for bacteria‐mediated metabolic adaptations of the snail ecotypes to their diet or other environmental factors, which will be in the focus of future metagenomic and functional studies.

### 4.2 The insight into the snail diet

*Littorina saxatilis* is a non‐selective grazer on the rocky surfaces, consuming mainly diatomes, cyanobacteria and detritus but it was also shown to feed on green algae, e.g. *Enteromorpha* (Berry, 1961; Fretter & Graham, 1962; Lotze & Worm, 2000; Otero‐Schmitt et al., 1997; Reid, 1996; Sacchi et al., 1977). Most of these organisms were found both in the biofilm samples and in the snail guts by 18S metabarcoding. Among the environmental samples, there was a difference in the biofilm composition in the high and low parts of the cliffs, as expected from a strong vertical zonation on the shore. In the snail gut content, there was a statistically significant effect of the ecotype on the eukaryotic DNA profiles by PERMANOVA. Two distinct profiles were found in the gut content of the Wave‐ecotype of *L. saxatilis* on the high shore and the Crab‐ecotype of *L. saxatilis* on the boulders; the profiles for the Wave‐ecotype of *L. saxatilis* on the low shore was in‐between the Wave – high shore and the Crab ecotypes.

The major components of rocky shore microphytobenthic biofilms are diatoms and cyanobacteria (Decho, 2000; Ginnever, 2014; Thompson et al., 2004), and we expected them to be in high abundances in the snail gut content. The diatoms were found to be the main diet in morphological analyses of the *L. saxatilis* gut content in Galicia (Otero‐Schmitt et al., 1997) and in the Venice lagoon (Sacchi et al., 1977), followed by cyanobacteria. In our 16S metabarcoding results cyanobacteria were also common in the snail guts. However, the diatoms were not the major component of the biofilms or of the snails' gut content by the 18S eukaryotic metabarcoding. Instead, we found DNA from many other groups of eukaryotic organisms, e.g. the red encrusted alga *Hildenbrandia*, ciliate and flagellate protists, small invertebrates and fungi. The microphotobenthic components of the biofilm (diatoms and cyanobacteria) produce the extracellular polymeric matrix that serves as a home for non‐phototrophic bacteria, protozoa, fungi, macroalgal germlings and invertebrate larvae (Decho, 2000; Toole et al., 2000). All these organisms can be potentially ingested by the grazing snails. Indeed, small crustaceans and nematodes have been previously found in the gut of *L. littorea* (Reid, 1996) and *L. saxatilis*, together with ciliate and flagellate protists (Berry, 1961; Sacchi et al., 1977); it is unknown however whether these organisms can be actually digested by the snails. Similar, *Hildenbrandia* and other red encrusted algae have been reported among the food spectrum of *L. littorea* (Reid, 1996); in the present study they appear to be a quite common in the guts of *L. saxatilis* from the lower shore.

The diet profiles of *Littorina* snails obtained here by metabarcoding are therefore somewhat different from the previous results based on the morphological observations, suggesting that other organism groups may be important food sources for *Littorina* in addition to diatoms, cyanobacteria and ephemeral green algae. However, the relative abundances of different eukaryotic organisms obtained by the bulk metabarcoding should be treated with caution, since low abundance or absence of a certain group in a sample may depend on the used metabarcoding primers. Since diatoms are known as a main component of intertidal epilithic biofilms (Decho, 2000; Ginnever, 2014; Thompson et al., 2004), their relatively low abundance in our samples may be due to a primer bias. For this first survey we chose universal 18S primers that are designed to cover major eukaryotic diversity (Wang et al., 2014), however they may have low affinity to specific groups. There have been recent developments of metabarcoding protocols for diatoms (Bailet et al., 2020; Groendahl et al., 2017). Future studies of the ecotypes' diet would benefit from these, in combination with morphological identification since none of the previous studies was conducted in the Swedish populations.

Despite the possible pitfalls of the metabarcoding approach, the overall patterns of eukaryotic diversity in our samples are strikingly similar to the patterns in the bacterial diversity. Interestingly, the gut content of the snails did not simply mirror their environment. Similarly, Norton et al. (1990) and Otero‐Schmitt et al. (1997) have found that the prevailing diatom species in the snail guts were not among the most common in the biofilm, suggesting that there may be some inadvertent selection due to the snails' ability to remove different diatoms from the surfaces. Reconstruction of the diet and the gut microbiome for a species feeding on the biofilm, such as *L. saxatilis*, presents a challenge since there may be many transient bacterial and eukaryotic microbes, inadvertently ingested and passed through the guts. Disentangling these will require combining molecular and morphological methods.

### 4.3 The potential role of microbes in the evolution of the snail ecotypes

Most examples of the host and gut microbiome collaboration in adaptation still come from mammals and insects (Douglas, 2014; Engel & Moran, 2013; Krajmalnik‐Brown et al., 2012; Ley et al., 2008). However, the metabarcoding techniques have recently opened up for studies of the microbes' role in the adaptation in marine organisms. For example, it has been shown that bacteria help the ascidian *Ciona intestinalis* to colonize new habitats by producing toxins, enhancing nitrogen recycling and providing resistance to heavy metals (Utermann et al., 2020). The subtidal and intertidal populations of the sponge *Hymeniacidon heliophila* have very different microbiome assemblages that contribute to the nitrogen cycling of the host (Weigel & Erwin, 2017). Symbiotic bacteria are the source for defense toxins for the bryozoan *Bugula neritina* (Lopanik et al., 2004) and probably for the sacoglossan mollusc *Elysia rufescens* (Davis et al., 2013).

Many studies of rapid ecological adaptation in *Littorina* focused on the genomics of divergence and on the two ecological factors that the snails adapt to, namely the crab predation and the wave exposure (Faria et al., 2019; Johannesson et al., 2017; Westram et al., 2018, 2021). The data is available on a number of genomic regions probably involved in adaptation, including chromosomal inversions, and a close correlation between the genomic variation and the environmental gradients. What is lacking at the moment is insights in the organismal functions behind the ecological adaptations in *Littorina*, and we argue that diet and gut microbiome composition may be an important and so far an over‐looked aspect.

Probably the most striking difference between the *L. saxatilis* ecotypes is their size: the adult snails of the Crab‐ecotype is at least twice bigger than the adult Wave‐snails (Janson, 1982; Johannesson, 1986; Westram et al., 2018). In addition, the ecotypes also differ by their shell shape (Le Pennec et al., 2017; Panova et al., 2006). In *L. littorea* the differences in the growth rate and even the shell shape have been shown to depend on the diet (Kemp & Bertness, 1984). Similarly, different growth rates in Pacific abalone *Haliotis discus hannai* is also associated with variation in the intestinal microbiota (Choi et al., 2021). The observed differences in size and growth rate of the two ecotypes imply the underlying differences in nutrients uptake, digestion and metabolism, which are on one hand depend on the diet and on the other hand may be faciliated and regulated by the gut microbiomes. Accordingly, there is often a close correlation between the diet and the composition of gut microbiota (Krajmalnik‐Brown et al., 2012; Ley et al., 2008).

Given the wide metabolic scope of the bacteria, they can potentially assist the adaptation and promote the divergence of the *L. saxatilis* ecotypes in many ways. One interesting biological function is hydrolysis of complex algal polysaccharides. In terrestrial herbivores the gut microbiome is responsible for the major part of the plant material breakdown (Flint et al., 2008). Much less is known about gut bacteria of marine herbivores; however, a recent study reported that *Vibrio* and *Psychromonas*, common in the gut of the abalone feeding on algae, have enzymes degrading cellulose, agarose, laminarin and alginate (Gobet et al., 2018). These bacterial genera were also among the most common gut symbionts in *Littorina* snails, found in the present study. Further, the ability to digest cellulose has been previously shown for other *Littorina* species (Norton et al., 1990); this ability could be provided by the gut bacteria.

Another important biological function that can be aided by the microbiome is calcification. Calcification and shell formation requires large energy resources in gastropods (Kemp & Bertness, 1984), and is one of the biological processes greatly affected by acidificaton and warming of the sea (Irie et al., 2013; Leung et al., 2020). Shell thickness is also among the characters separating the Crab and Wave ecotypes of *L. saxatilis* (Janson, 1982; Reid, 1996). In corals, another group of calcifiers, the calcification occurs with the help of symbiotic bacteria and dinoflagellates (Mouchka et al., 2010), and bacteria help the corals to maintain their calcification rate despite the environmental changes (Zhang et al., 2021). There are many other biological functions that are regulated by symbiotic bacteria in animals, that could also be relevant for the snails, such as immune response, synthesis of essential biological compound and vitamins, detoxication and so on.

There is a growing awareness that microbes and their hosts probably evolve together, including co‐adaptation of their genomes, so‐called “hologenome” concept (Bordenstein & Theis, 2015; Rosenberg & Zilber‐Rosenberg, 2016). Given the accumulated ecological and genomic knowledge on the evolution of the *L. saxatilis* ecotypes, this system has a great potential of developing to a hologenome model of rapid adaptations in marine organisms. We foresee the future advances coming from metagenome studies of the host‐associated bacteria and functional studies of the snails in combination with already existing genomic information for the host.

## CONFLICT OF INTEREST

The authors declare no conflict of interest.

## Supporting information


Figure S1

Figure S2

Figure S3

Figure S4

Figure S5

File S1

File S2

Table S1

Table S2

## Data Availability

Raw sequence data for this study are available at the NCBI archive, BioProject PRJNA778532.
