# Supplementary figures and images for: First insights into the gut microbiomes and the diet of the Littorina snail ecotypes, a recently emerged marine evolutionary model
Source: Evol Appl. 2022 Jul 24;16(2):365–78. doi: 10.1111/eva.13447 (PMC9923488; doi:10.1111/eva.13447)

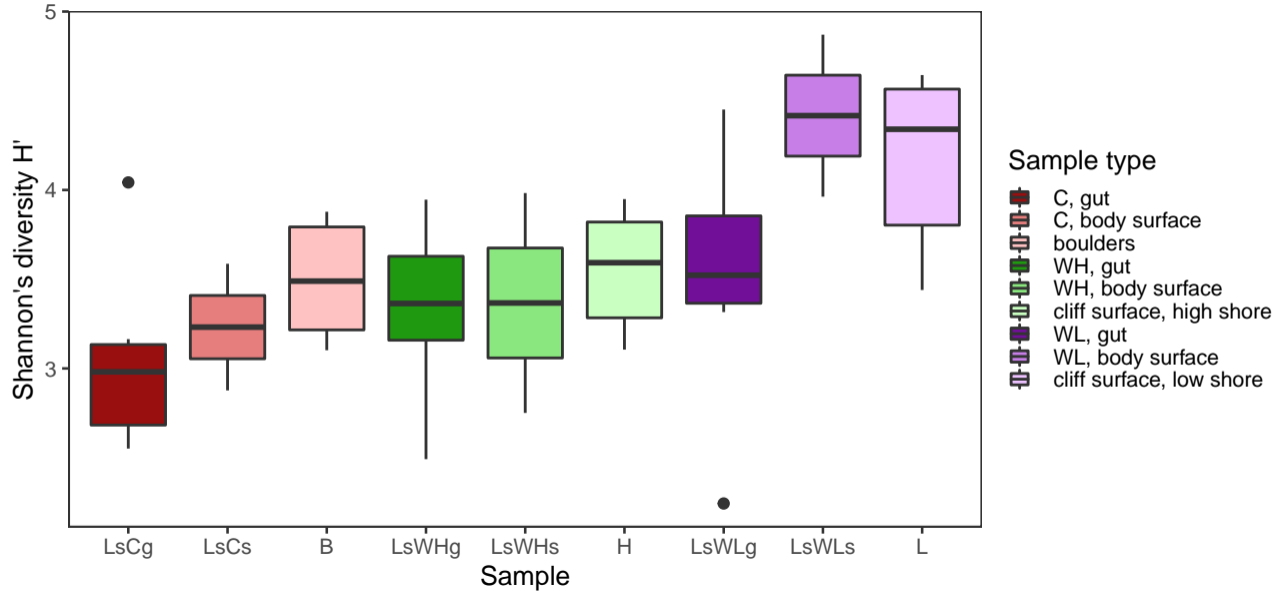

Supplement: Supplementary file 1 — Figure S1 [file EVA-16-365-s005.pdf]

**A** 16S: environment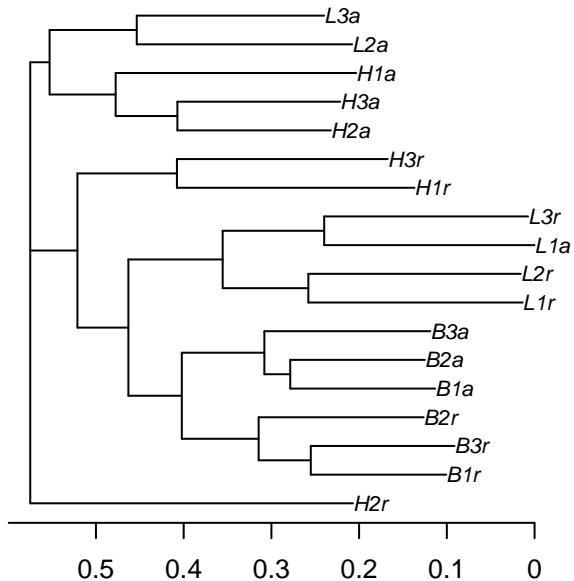**B** 16S: snail guts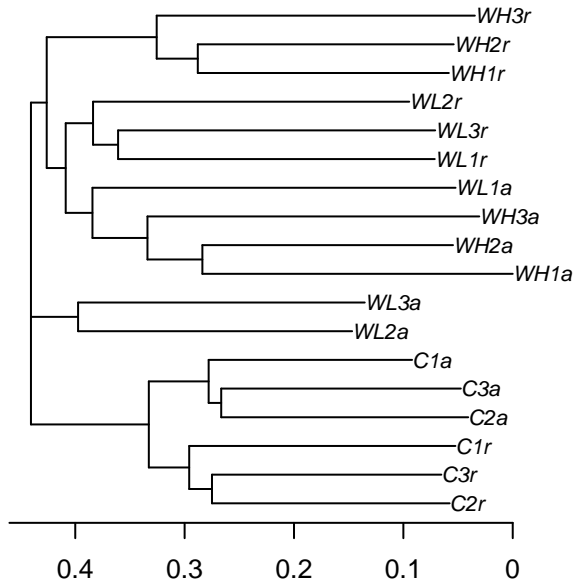

Supplement: Supplementary file 2 — Figure S2 [file EVA-16-365-s009.pdf]

**A**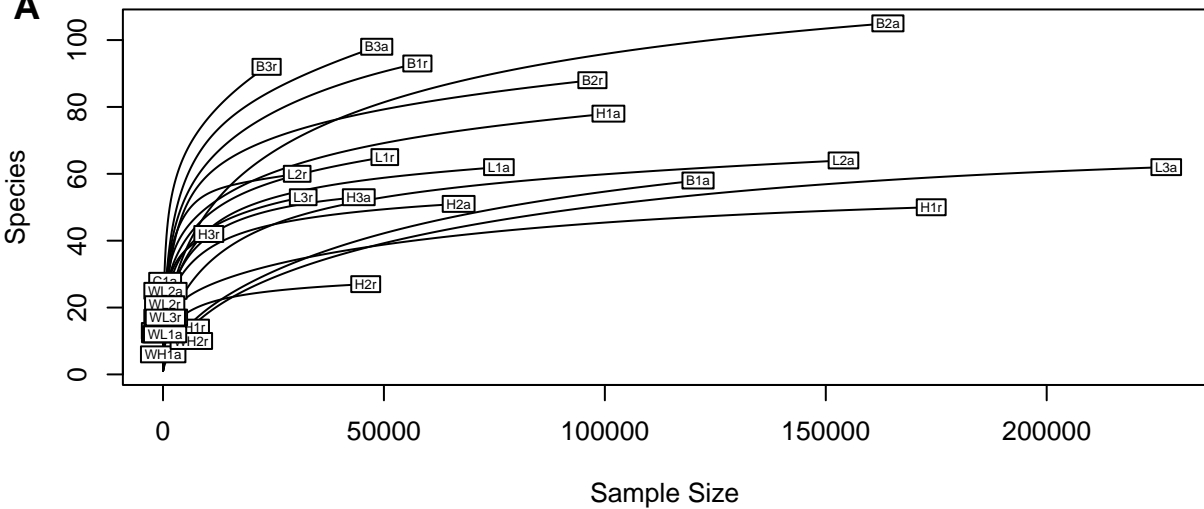**B**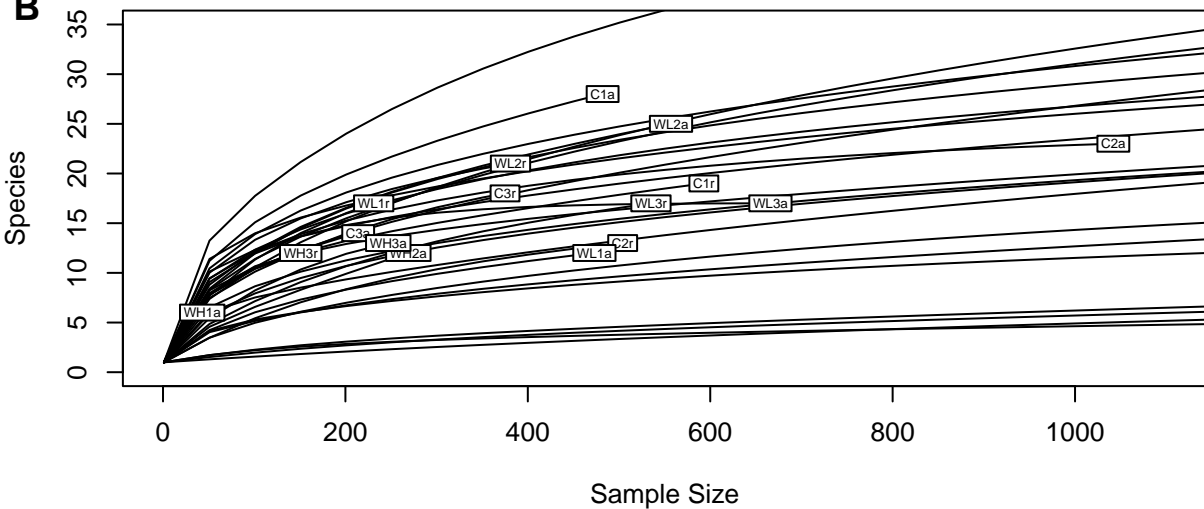

Supplement: Supplementary file 3 — Figure S3 [file EVA-16-365-s004.pdf]

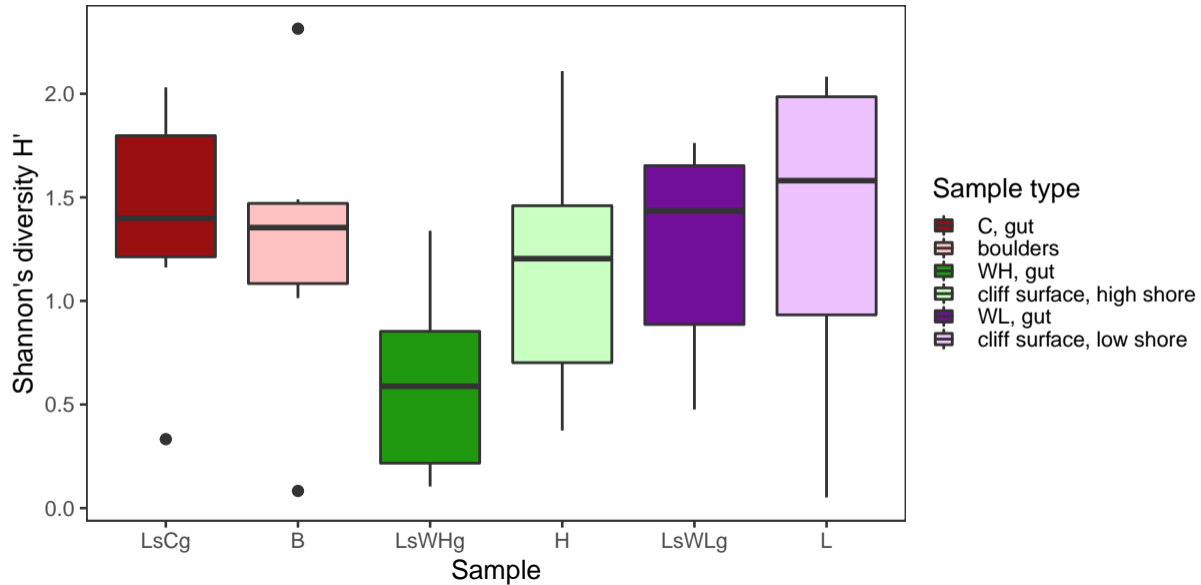

Supplement: Supplementary file 4 — Figure S4 [file EVA-16-365-s007.pdf]

**A** 18S: environment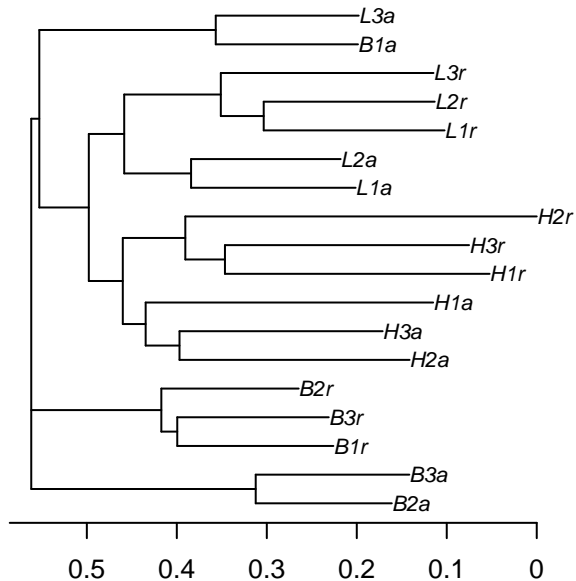**B** 18S: snail guts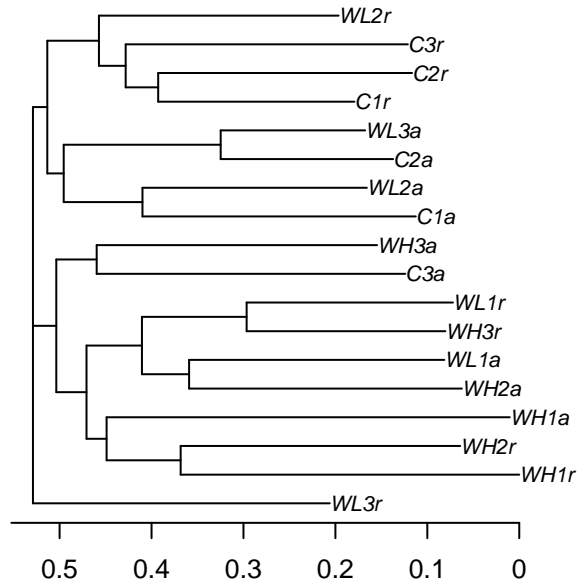

Supplement: Supplementary file 5 — Figure S5 [file EVA-16-365-s008.pdf]
