# Supplementary material for: First insights into the gut microbiomes and the diet of the Littorina snail ecotypes, a recently emerged marine evolutionary model
Source: Evol Appl. 2022 Jul 24;16(2):365–78. doi: 10.1111/eva.13447 (PMC9923488; doi:10.1111/eva.13447)
Supplement: Supplementary file 6 — File S1 [file EVA-16-365-s002.html]

Javascript must be enabled to view this page.

magnitude
magnitudeUnassigned

B
H
L
LsC
LsWH
LsWL

286313287292239890383576382888379192

286313287292239890383576382888379192

1072222414231922551104

1072222414231922551104

1072222414231922551104

1072222414231922551104

1072222414231922551104

5212121380232982596

5212121380232982596

5212121380232982596

5212121380232982596

4512118378022982586

34

4321710

405191104361118368

2232991921160

2222531959

2222531959

2222531959

14616151

14616151

14616151

213148090

213148090

147590

147590

2135

2135

1801917312817118

123319891736

123319891736

123319891736

71582971176

71582971176

71582971176

16123686

16123686

16123686

210157942067110178

210157942067110178

210157942067110178

210157942067110178

210157942067110178

824821491740

82811491740

82811491740

82811491740

8391061322

24243418

401

401

401

401

74562734934575538193131342153

5111573

5111573

5111573

5111573

74488734914556137313127442066

545567

545567

545567

71242991011113402267625483

71242991011113402267625483

21746841

185101

70742989310769352266725341

4815941

62701435723259431942844415059

205

205

5235216262374513412791419

3965

168

5235216261961213412791419

3093

797

2296

5746621946176730602716513640

195922148180498

184

5744721354158129122698513142

162

162

2830

2830

792

792

4560380832291103

4560380832291103

4560380832291103

9822209869414

141579235290

1115615199

3156362091

84263634124

84263634124

826

826

826

23479

23479

23479

23479

2113751398

2113751398

2113751398

2113751398

2346

2346

2346

2346

2346

6194

6194

6194

6194

6194

1227230241565

1227230241565

58692951542

58692951542

58692951542

6403731023

6403731023

6403731023

62144158622153979

3143241302556

3143241302556

241302556

4970122

86195264

9637121

1049

3143

3143

8723190

8723190

8723190

8723190

321125818903233

22179110742389

320

320

13723520

13723520

2117518192369

2117518192369

1467816844

25204

25204

1465296840

1452249794

134746

47351176543294537669013681

10293261889

10293261889

10293261889

10293261889

319281213153903583111942

4672119331181729

4672119331181729

4672119331181729

13042125757557288

1274179564520271

1274179564520271

31461933717

3143653411

312836

185209811792213409310925

185209811792213409310925

1656536545417797109

77214102406587

1514452503004461699

14715291355390465

1521210721065

114

114

114

114

3387895029886167451650

309176553170106

309176553170106

28854319305081

26333612025

3357791323334536661543

3357791323334536661543

3291302555614

58417212319840455

326431111248218464224

176

362165612107250

12013291

12013291

12013291

9513637612546852017600

9513637612546852017600

9513637612546852017600

9513637612546852017600

9513637612546852017600

1181116163923197946058159

12224112367

1088354

1088354

1088354

12123232013

12123232013

12123232013

500638234636912755798

500638234636912755798

500638234636912755798

500638234636912755798

913443541229231415

262717

262717

262717

893442914229231398

37691377153679

145861613

7244543

166954792663

522751537214195319

522751418214195319

119

57848901497030761879

2760

2760

2760

36272323

36272323

36272323

908647222631115

5114715

5114715

36931

36931

90859621941569

90791621917068

682451

2741556263773767

2741555976127

2741555976127

1159672708

1143390562

416067

1212279

74032

74032

21321053674

21321053674

21321053674

134581073930

134581073930

134581073930

1040942310541382143793843

1040942310541382143793843

1

1

1

100323699399369516242759

1102977116564196

1102977116564196

111502239

111502239

99113408478350815602524

5673356826134411831087

52541341

671663558437

285

201661131100

859441017901191271

66311377060688

3775477912527551081

3775477912527551081

3775477912527551081

3633

3633

3633

13581491631760676882710536745955

1314077687768845679959746342780

1118855823491103

52332344984

52332344984

7177539

7177539

157

157

17

17

1

1

42611

42611

11980

11980

1130474225318340479940717637

430231011

430231011

54144518983521

54144518983521

3624645940

161435

36854195

1120404180617595474640277565

19011096611461952571362

99155141899749313631184603

79924203641

3708432554595157455

71971607717137904471032

5311509101272

145712050034979312418810422338

6378934576189132

167223

44312395257

258866380116

7113313

2283

131181969230729309988765121528

70731482210062229785709812364

102692166

1792

103112

2135840253

6263651122744359

8610159

2966635

725254229

5074528545

140712987804395345

754122228

3110258121

19465610

76115692219

524

38184511119333

4315452133580404

391111234

241

745373227484543834

3398165620245634

348670020194693034

126222

35711615085137819

137

31272525

154152069

140

4222772

1746126

1279737

95173958534863236

1087420246

150115251717503571225

157

60712

212647717

137648025

2930320117

1394655376777404

1373661945255266

9418283

253251314135

1081

1081

6762541485568

6762541485568

160287205

160287205

355087876

355022647

355022647

65229

65229

914125141465225161270810364

84949

84949

1836

1836

48613

10213

384

892120831082040923592809

9423

2573220117

639354324425827856

1172444430

103241

2381160439955122041873

7476338233

58174

592

10

31902545847

18411

5836

36254

11237307246943497495

11237307246943497495

2829161063662281531262

2829161063662281531262

2829161063662281531262

440714754722283279043175

440714655722283279043171

440714655722283279043171

432711590672780578753118

992888

712137487192953

994

994

994

1452364

1452364

1452364

1452364

1452364

117161901438

117161901438

117161901438

117161901438

117161901438

1774401895969

7412559

7412559

741274

741274

485

485

1728189379

226

226

226

152189379

152189379

152189379

576289571993626226995213364237860

157714112041120186645162675143842

1431111521291

1431111521291

142112

1111401291

210395

210395

210395

18312376107407276236155

18312376107407276236155

11501132582

18012172104957067034916

148395229

31347565303

125

1118242

1118242

1118242

10321292621237

10321292621237

193115930

10319811947

109

639

639

639

92510

92510

92510

8210384930

8210384930

8210384930

27591522611226725683479129

474916

474916

913724531999941

913724531999941

164160040450219

164160040450219

14007950205111941555650

11371

1092

13997950180511911555650

9526683412832341977311

2629618532420933649

928611

1122278

156208445210618123406

63213

421374

7569901598192291241

150832891662

2418710

12511024

182262

246243015246

246243015246

984045591045116219982121

984045591045116219982121

984045591045116219982121

1906941722141743516

3513

3513

1002486301136

10030

247

2209301136

85183131165374211

6116121

601613420325186

16172033458

31111691417

249504191256296

190

151624

13418319632289

11307

46334222576177238274

17202042681197724

1811364

101120332419

7812281080641

29132182308165267550

29132182308165267550

29102081345690

236145737675

1155424466

233937

67214146

22236026

1155013

1155013

6362582

6362582

161253436627610441170

161253436627610441170

161253436627610441170

157

157

157

36566784242

36566784242

3282773921

376401321

713354

713354

713354

143075682

143075682

40

455

92872

775610

1605831989481548494882060450

1605831989481548494882060450

571347176985138706

317668220334331971718244

98115689111200862393540920

134154

24371281

76253495

7284115

7284115

7284115

112244

112244

112244

21512134

14734

14734

2136510

1122810

1137

104

104

104

28712648467797672475826

2341252278542168233147

23327424215212

12643557541

11239411963094

7111619

7111619

9927

9927

314281502444

3117855899

250951545

10911759

711759

102

5083012881957

5083012881957

5531

4051

148

13814182

13814182

1260263336

1260263336

1260263336

218199120

218199120

218199120

60548

60548

60548

56733964672

56733964672

56733964672

56733964672

418325459846062391244715186683

672

672

672

37855348038305

375121536305

375121536305

3

3

34513722

34513722

118612852315462104912146417079

118612852315462104912146417079

141335

1522211

396936821822139925921968

48122211258272434

1118187

2967285

115729264552366441

383679053156891366

147118864

112520154684133

211904119

1311596

21366187698

3481214053278

1477883329

323207662442121332188

3971319659

620321474108826562121019449

111116103103

3437546661556

20241117

332915494

32754278

32754278

3161216

3161216

956513918

956513918

94113918

524

3315203603517462470

54187598417402332

54187598417402332

40

40

241134625

241134625

4161773

4161773

90091427778012978691839381

90021412725512806545636919

1012414

367121794225226773648

21359118472347

86231289597810405265130766

11781158

110

110

33171144

33171144

73946208

73946208

141571711971967

141571711971967

1286974147

1286974147

301786

301786

1364116

1364116

1364116

241186380162823701896

16313197627589787

16313197627589787

333114293240252

333114293240252

45170697081541857

45170697081541857

2327201129811256118715789

2327201129811256118715789

2327201129811256118715789

4089723932214639120335249

4089723932214639120335249

3651118787840995423

94115084134309

361260461609276760122911

15747294248731594

31851001912

2339

238

238

2101

2101

142211510814270132312704002

98402631973312398673186

2115938520

82314252689

34356220491

35972413

59222532793910954501827

2102

2995475335453

3766781855247693

438112477453784403816

437212477453783380805

912311

25164446591422663

551802577

551802577

551802577

266

266

266

221172605833

2113595812

2113595812

20159121

1215911

8110

3161220

3161220

3161220

2500

2500

2500

34036

10

10

33036

33036

3150433

2398227

148221

22506

75226

460

1172

1756
