# Supplementary material for: First insights into the gut microbiomes and the diet of the Littorina snail ecotypes, a recently emerged marine evolutionary model
Source: Evol Appl. 2022 Jul 24;16(2):365–78. doi: 10.1111/eva.13447 (PMC9923488; doi:10.1111/eva.13447)
Supplement: Supplementary file 7 — File S2 [file EVA-16-365-s006.html]

Javascript must be enabled to view this page.

magnitude
magnitudeUnassigned

B
H
L
LsC
LsWH
LsWL

5117234421245693693210128282843

5117234421245693693210128282843

95885181986120765107311989403

38010125158748597211931288

340115

340115

1

339115

58421

58421

58421

35494125128738441511924123

33034759

33034759

351641251287384681192464

351641251287384681192464

1992121

1992121

1992121

27315212

267152

267152

621

621

2791426

2791426

2791426

4895555162

5555162

5555162

489

489

23241

23241

23241

12012

12012

12012

25

25

25

25

578295590111327510157115

577495570511326010056113

577495570511326010056113

4262531233

16421

675048

4748477480616891262

458254921632331114148

581699112

2587212

2587212

338271

338271

1266

1266

1266

211

211

211

449271

449271

439241

6

379241

1

1

3

3

40986624228939371114048081953

39264499471359325140187

39147495131358825140187

21654769213071443161

21654769213071443161

1182095

1182095

368641612512219726

368641612512219726

2

2

2

14325

14325

14325

116

116

116

4522571

4522571

4522571

4522571

37055719232038006113786681766

37055719232038006113786681766

11525797384821

8680584283612

1461

269921309129

1

1

37341

37341

35899419224137898213746601745

35899419224137898213746601745

389874034423770522466

456174952248172

456174952248172

456174952248172

1711843

1261127429

1451612

145184147

1738221

62322417

62322417

62322417

62322417

150

150

150

150

253135824396135621238

6224535723911130

710114

710114

211110

211110

2625

2625

133

133

35234935281120

319349521

3330261119

7816588190111

7816588190111

7816588190111

702505355711165107

69252814544

69252814544

39031111197

8531111197

305

8291324756

8291324756

931822655

931822655

14241523

14241523

10639431424

276051

6051

4

23

363109

13

233109

2982

2982

100037304

24

97637304

66231

66231

66231

755184022477139

181804

181804

181804

6465115611

3432521

3432521

226153

226153

77348211

77348211

91311097138

520902818

520902818

8111194220

8111194220

51

51

621726186

621726186

22

22

22

222

222

222

16

16

1

15

5

5

5

60124166

60124166

59

59615166

6147

6147

6147

6147

6147

1453104331048310915

5111061455455

12

12

12

2561021453453

2561021453453

128141421112

1208832331

81

1

1

1

252

252

252

142

142

4

12

40115712

40115712

40115712

3104632

7218

1910

26810195885123

26810195885123

48543614532

2723396071

21673835

2424111

143456943543

143456943543

771903044

771903044

2731171062110

2731171062110

267109105110

267109105110

6812

6812
